# *Drosophila* Models of Human Disease

**DOI:** 10.1155/2018/7214974

**Published:** 2018-08-30

**Authors:** Louise Cheng, Antonio Baonza, Daniela Grifoni

**Affiliations:** ^1^Peter MacCallum Cancer Centre, Victorian Comprehensive Cancer Centre Building, 305 Grattan Street, Melbourne VIC 3000, Australia; ^2^Centro de Biología Molecular “Severo Ochoa”, Universidad Autónoma de Madrid, Calle Nicolás Cabrera 1, 28049 Madrid, Spain; ^3^Department of Pharmacy and Biotechnology, University of Bologna, Via Selmi 3, 40126 Bologna, Italy

This special edition was assembled around the theme of how the fruit fly* Drosophila* is used as a disease model.* Drosophila* has been used productively as a model organism for over a century to study a range of diverse biological processes, including genetics and inheritance, embryonic development, organ regeneration, learning, behaviour, and aging. As most of the fundamental biological mechanisms and signalling pathways that control development and survival are conserved throughout evolution, there are many precedents that demonstrate the utility of using* Drosophila* as a model system. Mechanistic details of genetic and molecular regulation of cellular processes can be thus established in the fruit fly and then transferred to other organisms.

*Drosophila* research has produced numerous seminal discoveries for more than a century, which have translated into beneficial health outcomes, starting with Morgan's landmark discovery that genes are carried on chromosomes, which has underpinned modern genetics. The striking observation that around 75% of the genes responsible for human diseases are evolutionarily conserved across animal species, including* Drosophil*a, has meant that the study of this organism has facilitated the understanding of multiple aspects of an increasing number of human diseases. Furthermore, the ability to perform sophisticated genetics with large progeny numbers and fast-generation time has allowed scientists to define the molecular mechanisms of gene function at a level of resolution and rapid pace not achievable with other animal models. We have selected several areas to cover in this issue, including recent advances in the understanding of the mechanisms controlling organ regeneration, neurodegeneration, cancer, and metabolic diseases.

Many neurodegenerative diseases are caused by different defects in posttranslational modification or build-up of aberrant proteins. We have included two reviews and three studies which report and discuss the use of* Drosophila* to model neurodegenerative diseases and possible therapeutic approaches. D. Denton and L. O'Keefe highlight how autophagy and defects in lysosome-mediated degradative pathways contribute to the etiology of Alzheimer's Disease and how this can be modelled using* Drosophila*. M. D. Moltó and J. V. Llorens focus on how* Drosophila* models have gained new insights into the involvement of lipid metabolism and glial cells in Friedreich's ataxia, caused by a deficit in the mitochondrial protein Frataxin. U. Mayor et al. present how the ubiquitination state of neural proteins and ubiquitin carriers can play important roles during neuronal development and in disease setting, and M. Jafari et al. show how some natural compounds may help contrast a number of neurodegenerative traits in Huntington's and Alzheimer's diseases.

Cancer is driven by complex genetic and cellular mechanisms. The use of* Drosophila* as a model to study cancer has proven essential to elucidate several mechanisms that are fundamental to cancer development. Here, we have included three reviews on how* Drosophila* is utilised to dissect the mechanisms underlying tumourigenesis and cancer progression. L.-A. Baena-López et al. present an overview on the function of caspases, independent of their traditional role in cell death, in contexts such as proliferation, differentiation, and migration, all of fundamental importance in tumourigenesis. J. B. Cordero et al. use the* Drosophila* midgut to interrogate cell autonomous, niche-derived signals which coordinately regulate stem cell proliferation in response to tumourigenesis. Finally, H. E. Richardson and M. Portela give a comprehensive update on cooperative oncogenesis, where multiple mutations/genetic alterations cooperate to drive tumourigenesis, which can be beautifully recapitulated in* Drosophila*.

Regeneration is an amazing ability displayed in different forms by most metazoans, including humans, which allows organisms to repair or totally replace damaged organs. Given the potential therapeutic applications of understanding the mechanisms that control this capacity, in recent years a lot of effort has been focused on analysing the genetic and molecular basis of this intriguing phenomenon. In this issue, A. Baonza and S. Ahmed-de-Prado review the contribution of* Drosophila* to identify the signalling networks involved in regulating the variety of cellular responses required for regeneration.

C. Gamberi et al. and M. Mink et al. present novel* Drosophila* models of human kidney disease and explain how studies using the malpighian tubules can shed light on cyst formation in kidney disease and the importance of some basement membrane components in the context of nephropathy. Finally, J. R. Riesgo-Escovar et al. present an overview on how* Drosophila* is being increasingly appreciated as a model for human metabolic diseases. Although the physiology of insects is different from that of mammals, the genes and signalling pathways involved in growth control are highly conserved.* Drosophila* with diabetic and obese phenotypes can be generated by manipulations of pathways that cause parallel clinical manifestations in humans, further validating* Drosophila* as a model for studying human physiology and metabolism. In this sense, P. Bellosta et al. propose a* Drosophila* model of Adipose Tissue Macrophage (ATM) infiltration to study the protective role of anthocyanins in chronic tissue inflammation and related metabolic diseases.

Taken together, these reviews and studies highlight the continuous innovation in the use of* Drosophila* for the study of disease mechanisms, which has been revealing many insights in regard to human disease onset, phenotypes, and progression.

*Louise Cheng* *Antonio Baonza* *Daniela Grifoni*

